# Healing Effect of Vicenin-2 (VCN-2) on Human Dermal Fibroblast (HDF) and Development VCN-2 Hydrocolloid Film Based on Alginate as Potential Wound Dressing

**DOI:** 10.1155/2020/4730858

**Published:** 2020-04-23

**Authors:** Woan Sean Tan, Palanisamy Arulselvan, Shiow-Fern Ng, Che Norma Mat Taib, Murni Nazira Sarian, Sharida Fakurazi

**Affiliations:** ^1^Laboratory of Vaccines and Immunotherapeutics, Institute of Bioscience, Universiti Putra Malaysia, 43400 Serdang, Selangor, Malaysia; ^2^Muthayammal Centre for Advanced Research, Muthayammal College of Arts and Science, Rasipuram, Namakkal, Tamil Nadu 637408, India; ^3^Scigen Research and Innovation Pvt Ltd, Periyar Technology Business Incubator, Periyar Nagar, Thanjavur, Tamil Nadu 613403, India; ^4^Centre for Drug Delivery Research, Faculty of Pharmacy, Universiti Kebangsaan Malaysia, Jalan Raja Muda Abdul Aziz, 50300 Kuala Lumpur, Malaysia; ^5^Department of Human Anatomy, Faculty of Medicine and Health Sciences, Universiti Putra Malaysia, 43400 Serdang, Selangor, Malaysia

## Abstract

Chronic wounds represent serious globally health care and economic issues especially for patients with hyperglycemic condition. Wound dressings have a predominant function in wound treatment; however, the dressings for the long-lasting and non-healing wounds are still a significant challenge in the wound care management market. Astonishingly, advanced wound dressing which is embedded with a synthetic drug compound in a natural polymer compound that acts as drug release carrier has brought about promising treatment effect toward injured wound. In the current study, results have shown that Vicenin-2 (VCN-2) compound in low concentration significantly enhanced cell proliferation and migration of HDF. It also regulated the production of pro-inflammatory cytokines such as IL-6, IL-1*β*, and TNF-*α* from HDF in wound repair. Treatment of VCN-2 also has facilitated the expression of TGF-1*β* and VEGF wound healing maker in a dose-dependent manner. A hydrocolloid film based on sodium alginate (SA) incorporated with VCN-2 synthetic compound which targets to promote wound healing particularly in diabetic condition was successfully developed and optimized for its physico-chemical properties. It was discovered that all the fabricated film formulations prepared were smooth, translucent, and good with flexibility. The thickness and weight of the formulations were also found to be uniform. The hydrophilic polymer comprised of VCN-2 were shown to possess desirable wound dressing properties and superior mechanical characteristics. The drug release profiles have revealed hydrocolloid film, which is able to control and sustain the VCN-2 released to wound area. In short, hydrocolloid films consisting of VCN-2 formulations are suitably used as a potential wound dressing to promote restoration of wound injury.

## 1. Introduction

Wound healing is a well-programmed complex process which involves inflammation, cell migration and proliferation, and neovascularization extracellular matrix production and remodeling [1]. The healing of chronic wounds still remains a significant clinical problem nowadays due to improper mechanisms that have response to repair the injured wounds. Chronic wounds have represented that a global burden leads to the impairment of quality of life and has a major impact on incurring a huge cost for treatment.

Wound dressings form a key part of wound management to achieve rapid wound closure and minimal acceptable scar [2]. Medicinal wound dressing is an effective approach for reaching an optimum milieu for wound healing through impregnated therapeutic drugs of different pharmacological action [3, 4]. Dressings cast from hydrocolloid film are the main choices among the popular wound dressings owing to their high absorption ability via strong hydrophilic gel formation characteristics [5]. Hydrocolloid is able to absorb exudates and form gel to provide a moist environment which is essential for healing. The simple, thin with semi-permeable films also permit for inspection wound condition, water vapor, and gases exchange for effective restoration of wound [6].

Alginate is a natural polysaccharide found in the cell wall of brown algae and possesses high gel-forming properties and biodegradable and biocompatible characteristics. It is a hydrophilic soluble salt of alginic acid which composed of two uronic acids, which is *β*-(1-4)-linked D-mannuronic acid (M) and *α*-(1-4) linked L-guluronic acid (G), and is made up of homopolymeric blocks M–M or G–G, and with an alternating sequence of M–G blocks. In a drug delivery system, alginate is usually exploited as drug-controlled release vehicle due to the ability to swell when in contact with water. This is important to control the slow release properties of the bioactive material from the gel to ensure the efficacy of the formulation [2, 7].

Relevance targets understanding effect of natural products in wound healing has attracted greater attention from the medical profession because it will further lead to more optimism in discovering efficient treatments with low side effects. Vicenin-2 (VCN-2) is a bioactive compound and flavonoid glycoside from a subgroup of phenolics which is known as apigenin-6,8-di-C-*β*-d-glucopyranoside. It has been reported to possess prospective antidiabetic, antioxidant, and anti-inflammatory properties and enhance cell proliferation and migration effect [8–10]. A previous study has identified the presence of VCN-2 in the bioactive fraction isolated from *Moringa oleifera* leaves. It was found to have a good wound healing ability in rats included with hyperglycemic condition. The extract was also found to have antimicrobial properties which have intensity to enhance the healing of a wound [10, 11].

In the present study, we have evaluated the wound healing effect of the VCN-2 synthetic compound through *in vitro* human dermal fibroblast (HDF). Assessments of inflammatory cytokines (IL-6, IL-1*β*, TNF-*α*) level and mediators (iNOS, COX-2, NF-*κ*B) expression played critical roles at the inflammation phase; cell proliferation, migration, and growth factor (VEGF, TGF-*β*) proteins acted important actions at proliferation phase of healing were conducted via ELISA and immunoblotting assays. Besides, a hydrocolloid film is aimed to develop and characterize for the intent to extend the period of drug release, prolong retention of dosage form, and enhance the VCN-2 absorption without much interference in the healing activities. The developed films are evaluated through its physico-chemical properties including mechanical strength, rheology, expansion rate, moisture vapor transmission rate (MVTR), and drug release profile. This study offers an alternative new formulation in wound therapy and is especially useful to facilitate wound healing in hyperglycemic condition.

## 2. Materials and Methods

### 2.1. Materials

Dulbecco's modified Eagle's medium (DMEM), penicillin/streptomycin antibiotics, fetal bovine serum (FBS), triple, cell counting kit-8 (CCK-8) reagent, bovine serum albumin (BSA), and radioimmunoprecipitation assay (RIPA) buffer for purpose cell culture and protein collection were purchased from Nacalai (Kyoto, Japan). The 3-(4,5-dimethylthiazol-2-yl)-2,5-diphenyltetrazolium bromide (MTT) was obtained from Sigma–Aldrich (St. Louis, MO, USA).

Bicinchoninic acid (BCA) assay was obtained from Thermo Fisher Scientific (Waltham, MA, USA). ELISA was bought from R&D System (Minneapolis, USA). Primary antibodies specific to VEGF, TGF-1*β*, iNOS, COX-2, NF-*κ*B, or *β*-actin were purchased from Santa Cruz Biotechnology (Santa Cruz, CA). The anti-rabbit or anti-mouse secondary antibodies conjugated to horseradish peroxidase were also obtained from Santa Cruz biotechnology (Santa Cruz, CA). Methanol, isopropyl alcohol, and dimethyl sulfoxide (DMSO) were used for immunoblotting process and acted as solvent to dissolve commercial compounds, respectively.

Sodium alginate (CAS No. 9005-38-3, Mw ∼ 20–40 kDa) and gelatin powder (bovine skin, type B) were obtained from Sigma–Aldrich Co. (St. Louis, MO, USA). Allantoin and Vicenin-2 (VCN-2) were purchased from Fluka Analytical (Sigma Aldrich, Germany). Propylene glycol 4000 in liquid state (purity >97%) and glycerol were supplied by R&M Chemicals (UK). Calcium chloride and potassium chloride were provided by Merck (Germany). All other chemicals were of analytical grade and distilled water was used throughout the experiment.

### 2.2. Cell Culture for Human Dermal Fibroblast (HDF)

Human dermal fibroblast (HDF) cells were obtained from the American Type Culture Collection (ATCC, VA, USA) that have been thawed and maintained in DMEM supplemented with 5% heat-activated FBS and 1% penicillin/streptomycin at 37°C in a humidified incubator with 5% CO2. The used passage of cell throughout experiment is not more than 25, and the medium of cells were changed every 2–3 days. Cells at 80–90% confluent condition were used for seeding and treatment or passaged by trypsinization. Extra healthy and growing cells were stocked in liquid nitrogen cylinder to maintain their exponential growth stage.

### 2.3. Cell Cytotoxicity (MTT) Colorimetric Assay

The cytotoxicity effect of synthetics compound VCN-2 was tested on HDF cells through MTT assay. The 100 *μ*L of HDF cells were seeded in triplicate into 96-well plates (1 × 10^4^ cells/well) and incubated for 24 h. The cells were treated with various gradient concentrations of VCN-2 compound with serial dilutions ranged from 6.25 to 400 *μ*M and then incubated for 24 h. Briefly, after 24 h treatment, 20 *μ*L of MTT solution (5 mg/mL in phosphate-buffered solution (PBS)) were added to each well and then follow by incubation for another 3 h. The medium was removed and the purple formazan crystals formed were dissolved by adding 100 *μ*L DMSO. The plate was swirled gently to mix well and kept in dark condition at room temperature for 30 min. The absorbance was determined by using a microplate reader at 570 nm wavelength. The results were expressed as a percentage of surviving cells over control cells.

### 2.4. Proliferation Assay

The proliferation effect of bioactive compound VCN-2 was tested on HDF cells by using cell CCK-8 reagent. 100 *μ*L of HDF cells suspension with (0.5 × 10^4^ cells/well) were dispensed in triplicate into 96-well plates and incubated for 24 h. The cells were then treated with VCN-2 with serial dilution at concentration 6.25, 12.5, 25, 50, 100, 200, and 400 *μ*M and incubated for 24 h in an incubator. 10 *μ*L of CCK-8 reagent was added to each well and further incubated for 3 h. The absorbance was determined by using ELx800™ Absorbance Microplate Reader (BioTek Instruments Inc., Vermont, USA) at 450 nm wavelength. The results were expressed as a percentage of surviving cells over control cells.

### 2.5. Scratch Migration Wound Healing Assay

The effect of VCN-2 on HDF cells migration was examined through scratch wound healing test. According to Fronza et al. [12] and Muniandy et al. [13], 300 *μ*L of cells suspension (2 × 10^5^ cells/well) was equally distributed into 24-well plates and incubated for 24 h. All media were aspirated and the monolayer HDF cells were scrapped horizontally with P200 micropipette tips. The cells were extensively rinsed by PBS to remove cellular debris. The medium was freshly replaced with the absence or presence of VCN-2 and allantoin at different concentration in new media. Photographs of wounded area were taken at 0 and 24 h through life cell imaging microscope at 40× magnification (Olympus,Tokyo, Japan). The images were analyzed using Image J software (Bio Techniques, New York, USA). Percentage of wound closure area and distance between cells were measured and compared with the value obtained during 0 h. Increment of the percentage of wound closure rate and reduction in length of the scratch distance indicated the migration and proliferation of HDF cells.

### 2.6. Determination of Cytokines (IL-1*β*, IL-6, and TNF-*α*) Level via ELISA

HDF cells without any drug treatment or with the treatment of VCN-2 synthetic compound at concentrations 12.5–100 *μ*M and allantoin (50 *μ*g/mL equally to 316 *μ*M) were seeded in 6-well plates (5 × 10^5^ cells/well) and incubated for 24 h. Cells with treatment of allantoin acted as positive control while cells without treatment were used as negative control for experiment comparison. The concentration of pro-inflammatory cytokines such as IL-6, IL-1*β*, and TNF-*α* were assayed in cultured media of HDF cells using human ELISA kits (R&D Systems Inc., MN, USA) according to the manufacturer's instructions. The optical density of each well was determined using microplate reader at 450 nm wavelength.

### 2.7. Detection of Proteins Expression (iNOS, COX-2, NF-*κ*B, VEGF, and TGF-*β*) via Western Blot

HDF cell protein extracts were harvested and prepared by using RIPA buffer for western blot analyses. The concentration of protein was determined by using bicinchoninic acid (BCA) assay according to the manufacturer's protocol. An equal amount of cellular proteins (20 *μ*g) were loaded onto 10% sodium dodecyl sulfate-polyacrylamide (SDS-PAGE) gel electrophoresis under reducing conditions (incubated with 5× laemmli buffer) for separation. The separated proteins in gel were then transferred to polyvinylidene difuoride (PVDF; GE Healthcare) membrane and was allowed to set for 1 h. The membrane underwent blocking for 1 h with the use of a blocking solution (5% of BSA in phosphate-buffered saline containing 1% Tween-20 (PBST)) at room temperature prior to incubation of specific primary antibodies such as VEGF, TGF-1*β*, iNOS, COX-2, NF-*κ*B, or *β*-actin at 4°C overnight. The membrane was washed 5 times with PBST followed by incubation with respective anti-rabbit or anti-mouse secondary antibodies conjugated to horseradish peroxidase for 1 h. It was followed by washing 5 times with PBST for 10-min intervals.

The bands were visualized under chemiluminescence system (Chemi Doc, Bio Rad, USA), followed by Image J software analysis.

### 2.8. Pre-Formulation Characterization of Blank Hydrocolloid Films with Different Percentage of Sodium Alginate (SA)

Solvent casting method was used to synthesize the drug-free films based on alginate. The alginate hydrocolloid films were first cast from the gel formulation which has been documented in Table 1. Formulation S1–S6, with varying amount of sodium alginate (10, 8, 6, 4.5, and 4% (w/w)), propylene glycol (15% (w/v)), and glycerol (6% (w/v)) or gelatin (4% (w/w)) were used to prepare film accordingly. To this end, 20 g of the uniform gel slurry was poured into petri dish with an area of 58.05 cm^2^ and then dried in oven at 45°C for 48 h. Finally, all the films were collected and underwent evaluation. The developed films have been compared based on physical characteristics such as appearance.

### 2.9. Preparation of Hydrocolloid Film Containing VCN-2

From the pre-formulation studies, S6 was characterized based on its physical characteristics such as smooth surface and no bubbles. It was selected to further produce drug-loaded films (H1–H4) which are presented in Table 2. H1 was SA base blank hydrocolloid film which acted as control. H2 and H3 were hydrocolloid films loaded with concentrations 12.5 and 25 *μ*M of VCN-2, respectively. H4 film was impregnated with standard allantoin drug with concentration 316 *μ*M as positive control. Firstly, the 4 g of gelatin (4%) powder was dissolved in distilled water and added 4.5 g of SA (4.5%) with constant stirring until uniform gel slurry formed at temperature 40°C. The propylene glycol (15%) with 15 g was then added into the gel followed by the desired compounds concentration and stirred continuously. Finally, 6 g of glycerol (6%) was further added into the hydrocolloid gel to stand at room temperature until all air bubbles were eliminated. Then, the 20-g SA hydrocolloid gel was poured into a petri dish (area 58.05 cm^2^) and dried in an oven at 45°C for 48 h. All the dried films were eventually stored in a desiccator for at least 48 h before proceed for further studies.

### 2.10. Measurement Thickness, Solvent Loss of Developed Film

Five positions (one at the middle, four near the edges) of each hydrocolloid film was measured to get the average thickness by using digital caliper (Messzeuge, Germany). The solvent loss is defined as the difference between the weight of film before and after drying base on following Equation (1). The data for all measurements were presented as mean ± SEM and performed in triplicate.

(1) $$\begin{array}{c} \text{Solvent loss}\left(\text{g}\right)=\text{Weight of hydrogel before drying}\left(\text{g}\right)–\text{Weight of hydrocolloid film after drying}\left(\text{g}\right). \end{array}$$

### 2.11. Measurement of pH of Developed Film

Digital pH meter (Mettler Toledo, Fisher Scientific UK) was used to determine the acidity condition of the hydrocolloid film development. 0.1 g of film was dissolved in 10 mL of distilled water and stored for 2 h. The measurement of pH value was performed in triplicate.

### 2.12. Determination of Moisture Vapor Transmission Rate (MVTR)

The dried hydrocolloid film was cut into a disc shape with a diameter of 22 mm and fixed over a dry vial that contains 3 g of calcium chloride as a desiccant. The circular film with a defined exposed diameter of 11 mm was sealed to the cap using a rubber ring and then mounted onto the brim of the vial. The vial was then weighted and kept in a desiccator containing saturated potassium chloride solution which to provide 84% relative humidity (RH) at temperature 25°C. The gain in the weight of each vial was measured every hour for a period of 8 h, followed by 24 and 48 h. The following Equation (2) was used to determine the MVTR for each formulation. The test was performed in triplicate for each formulation and data were obtained in mean ± SEM, (*n* = 3).

(2) $$\begin{array}{c} \text{MVTR}=\text{W}/\text{ST}, \end{array}$$

where W is the gain in weight of the desiccant (g), T is the period hours for experiment (h), and S is the exposed surface area (m^2^) of the film.

### 2.13. Examination of Film Expansion from Developed Film

Gelatin gel (4%) was used to simulate the wound surface. Four grams of gelatin powder was added into 100 mL distilled water at temperature 90°C and stirred constantly to get a clear solution. Gelatin solution with 20 g was then poured into a petri dish and placed at 25°C overnight to form a gel. All hydrocolloid films were trimmed into a circular-shaped disc with defined diameter 22 mm then fixed at the center of gelatin gel. All the changes in the diameter of the film was measured at the predetermined time intervals. The expansion percentage of each film was evaluated based on Equation (3). Three independent measurements were run and average values were obtained in the analysis test.

(3) $$\begin{array}{c} \text{Expansion}=\left(\text{Dt}-\text{Do}\right)/\text{Do}\times 100, \end{array}$$

where Dt is the diameter of the film after expansion and Do is the diameter of the film before expansion.

### 2.14. Mechanical Profile of Developed Film

Intron universal testing machine (model 5567, USA) was used to measure the tensile strength (TS) and the percentage elongation at break (%E) of the hydrocolloid films. According to Rezvanian et al. [14], prior to analysis, the sample specimen of films was conditioned at 25°C, 50 ± 5% RH for 48 h. Hydrocolloid film was cut into 30 mm for length and 5 mm for width dumbbell-shaped strips by using ASTM standard dumbbell-shaped template and cutter press (GT-7016-A Gotech testing machine, Malaysia). The specimens were undergoing stretching at a crosshead speed of 5 mm/min to their breaking point for tensile properties investigation. Typically, triplicated measurement was run for each type formulation. TS is defined as Equation (4) while %E is well-defined as Equation (5). The result is presented as average mean ± SEM.

(4) $$\begin{array}{c} \text{TS}\left(\text{MPa}\right)=\left(\text{Maximum load at break}\left(N\right)\right)/\left.\text{Thickness}\times \text{width}\left({mm}^{2}\right)\right), \end{array}$$

(5) $$\begin{array}{c} \%\text{E}=\left(\text{Lt}-\text{Lo}\right)/\text{Lo}\times 100, \end{array}$$

where Lt is the length of the film after undergo stretching and Lo is the length of the film before stretching.

### 2.15. Investigation Rheological Profile of Developed Film

Bohlin Gemini II rheometer with cone-and-plate geometry was used to access the flow properties of hydrocolloid gel (before drying) and rehydrated hydrocolloid film. The rehydrated sample was prepared by introducing the amount of water lost into the film to reform the gel base on Equation (1). Then, 1 mL of the gel was put over the Peltier plate and rheological measurement was determined by cone with a diameter of 25 mm and an angle 1° at constant shear rate of 500 s^−1^ for 3 min. Basically, three replication tests were carried out and data were shown in mean ± SEM. The apparent viscosities of the films were obtained at a shear rate of the 500 s^−1^.

### 2.16. Determination of Drug Release from Developed Film

Franz diffusion cell (Permegar. Inc., USA) was used to probe the drug permeation study of VCN-2 released from the hydrocolloid films. The receptor cell was filled with phosphate buffer solution (PBS), pH 7.4 with constant 50 rpm agitation, and maintained in the temperature 37°C throughout the experiment. All films except for control with a diameter 22 mm were placed over the cellulose acetate membrane (pore size 0.45 *μ*m) and placed on the top of the receptor compartment with diameter 10 mm. Parafilm was used to wrap the cell cap and all the openings including donor top and receptor arm to prevent evaporation of PBS. During the experiment, 1 mL of the sample was withdrawn every hour for the first 8 h and finally at 24 h. An equal amount of new PBS was added for replacement. This will be followed by determined the concentration of VCN-2 release through UV spectrophotometer (Shimadzu 1800, Japan) at wavelength at 335 nm. The cumulative amount and drug release profile were plotted against time. The average values then were calculated and recorded (*n* = 3).

### 2.17. Statistical Analysis

All of the data were summarized from three independent experiments and were indicated as mean ± standard error mean (SEM). The *p* value was examined through one-way ANOVA followed by Tukey's multiple comparisons test using IBM with SPSS 20.0 software (SPSS Inc., Chicago, USA). *p* value < 0.05 was considered as statistically significant.

## 3. Results

### 3.1. Influence of VCN-2 Synthetic Compound on Cell Cytotoxicity

**Figure 1:**
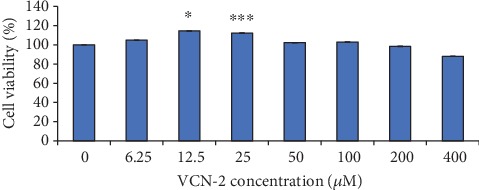
MTT assay was conducted to determine the toxic effect of VCN-2 synthetic compound on the viability of HDF cells. A density of 1 × 10^4^ cells/well of cells were seeded in 96-well plates and incubated with indicated concentrations of compound for 24 h. The data are presented as mean ± SEM of three independent experiments. There are statistically significant differences between VCN-2 treated groups versus normal control group at ^∗∗∗^*p* < 0.001 and ^∗^*p* < 0.05.

MTT reduction assay was conducted to access the toxic effect of VCN-2 compound on HDF cells. The cytotoxicity potential of VCN-2 was presented in Figure 1. The results showed that VCN-2 at lower concentrations have increased cell viability while at higher concentrations have reduced the cell viability. However, all dosages of VCN-2 used in this experiment did not exhibit any toxic effect to HDF cells since the cell viability was more than 80%. VCN-2 at concentrations of 12.5 and 25 *μ*M were the optimum dosages to be applied in HDF cells. Both concentrations significantly showed high percentage of cell viability, which were 114.40 ± 0.21% and 112.17 ± 0.16%, respectively, as compared to other groups.

### 3.2. Effects of VCN-2 Synthetic Compound on Cell Proliferation

**Figure 2:**
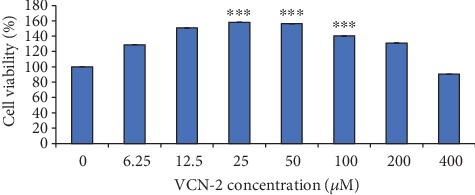
Effects of VCN-2 synthetic compound on the proliferation rate of HDF cells. A density of 0.5 × 10^4^ cells/well of HDF cells were seeded in 96-well plates and incubated with indicated concentrations of synthetic compound for 24 h. Cell viability was determined by CCK-8 reagent. The data are presented as mean ± SEM of three independent experiments. The baseline of normal control which was medium without treatment was set as 100%. There is a statistically significant difference between normal control group and VCN-2 treated groups at concentrations of 25, 50, and 100 *μ*M, ^∗∗∗^*p* < 0.001.

In this study, CCK-8 kit reagent was used to investigate the proliferation effect of VCN-2 synthetic compound at varying concentrations: 6.25–400 *μ*M on HDF cells. As shown in Figure 2, the presence of VCN-2 has increased cell viability of HDF cells except the group treated with VCN-2 at concentration 400 *μ*M with cell viability 90.45 ± 0.07%. The synthetic compound enhanced cell proliferation in a dose-dependent manner up to 50 *μ*M with cell viability of 156.66 ± 0.18%. The optimum stimulatory dosages at concentrations 12.5, 25, 50, and 100 *μ*M were applied for subsequent analysis with corresponding cell viabilities 150.73 ± 0.11%, 158.18 ± 0.16%, 156.22 ± 0.18%, and 140.33 ± 0.08%.

### 3.3. Influence of VCN-2 Synthetic Compound on Cell Migration

**Figure 3:**
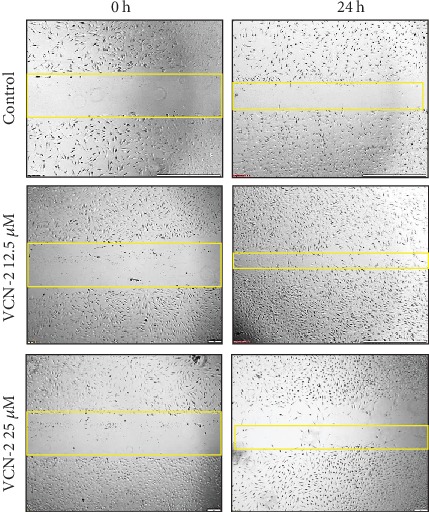
Microscopic image to evaluate *in vitro* wound healing effect of VCN-2 with scratch assay using a confluent monolayer HDF cells, with 12.5 and 25 *μ*M of VCN-2. Cell migration into the wound was observed in response to an artificial injury. A single representative area is shown immediately after 24 h the wounding with VCN-2 and allantoin treatments.

**Figure 4:**
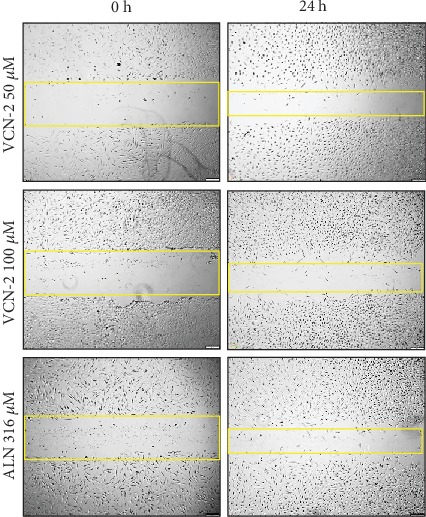
Microscopic image to evaluate *in vitro* wound healing effect of VCN-2 with scratch assay using a confluent monolayer treated with 50, 100 *μ*M of VCN-2 and 316 *μ*M of ALN. Cell migration into the wound was observed in response to an artificial injury. A single representative area is shown immediately after 24 h the wounding with VCN-2 and allantoin treatments.

**Figure 5:**
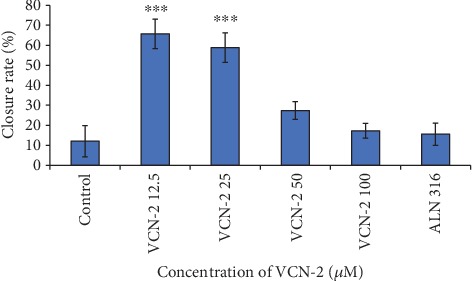
Potential wound closure rate of VCN-2 on HDF. The triplicate data are presented as mean ± SEM. There is statistically significant difference between normal control group and VCN-2-treated groups at concentrations of 12.5, and 25 *μ*M, at ^∗∗∗^*p* < 0.001.

The activity and influence of VCN-2 at dosages of 12.5, 25, 50, and 100 *μ*M on migration of HDF cells were investigated using the scratch wound healing assay (Figures 3 and 4). This assay was conducted to estimate the migration ability of HDF cells in the presence of VCN-2. Cellular migration of HDF was studied after 24 h of incubation of VCN-2 from concentrations 12.5 to 100 *μ*M. In Figure 5, the results showed that low concentrations of VCN-2 at 12.5 and 25 *μ*M have significantly induced the cell migration (*p* < 0.001) by 65.72 ± 7.4% and 58.86 ± 7.4%, respectively. In contrast, higher concentrations of VCN-2 at 50 and 100 *μ*M induced lesser migration and reduced wound closure rate of HDF cells with 27.43 ± 4.5% and 17.27 ± 3.7%, respectively. Allantoin that act as positive treatment showed wound closure rate with 15.58 ± 5.6%.

### 3.4. Effect of VCN-2 Synthetic Compound on Production of Inflammatory Cytokines (TNF-*α*, IL-6, and IL-1*β*)

**Figure 6:**
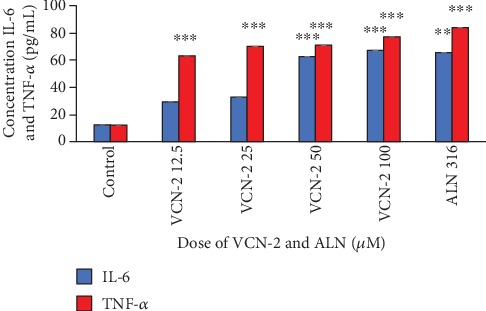
The effect of VCN-2 synthetic compound on the production of cytokines TNF-*α* and IL-6 on HDF cells. A density of 5 × 10^5^ cells/well of HDF cells were seeded in 6-well plates and treated with indicated concentrations of VCN-2 and allantoin for 24 h. Allantoin served as positive control. The supernatants were collected and analyzed by ELISA kits. The data are presented as mean ± SEM of three independent experiments. ^∗∗∗^*p* < 0.001 and ^∗∗^*p* < 0.01 indicated significant difference in VCN-2 and allantoin-treated groups versus the normal group.

**Figure 7:**
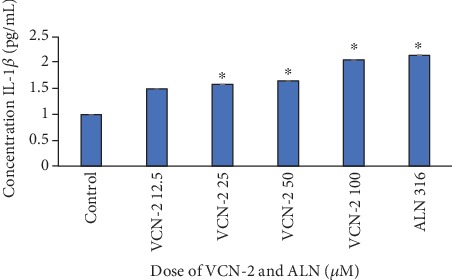
Detection IL-1*β* concentration on HDF cells. A density of 5 × 10^5^ cells/well of HDF cells were seeded in 6-well plates and treated with indicated concentrations of VCN-2 and allantoin for 24 h. Allantoin served as positive control. The supernatants were collected and analyzed by ELISA kits. The data are presented as mean ± SEM of three independent experiments. ^∗^*p* < 0.05 indicated significant difference in VCN-2 and allantoin-treated groups versus the normal group.

A series of pro-inflammatory cytokines including TNF-*α*, IL-6, and IL-1*β* was not only produced by polymorphonuclear leukocytes and macrophages but was also observed in some resident cell types such as fibroblast. The effect of VCN-2 synthetic compound on the production of these pro-inflammatory cytokines by HDF cells have been quantified through ELISA assays in the current study. Results in Figures 6 and 7 showed that treatment with VCN-2 and allantoin have significantly triggered the production of three types of cytokines in a concentration-dependent manner. As compared to control, treatment with 100 *μ*M of VCN-2 on HDF significantly increased the expression of TNF-*α*, IL-6, and IL-1*β* to 77.08 ± 0.002 pg/mL, 67.01 ± 0.012 pg/mL, and 2.04 ± 0.009 pg/mL, respectively. The levels of TNF-*α*, IL-6, and IL-1*β* have remarkably upregulated to 83.75 ± 0.007 pg/mL, 65.14 ± 0.009 pg/mL, and 2.13 ± 0.009 pg/mL after treatment with 316 *μ*M of allantoin.

### 3.5. Impact of VCN-2 Synthetic Compound on Expression of Inflammatory Mediators (NF-*κ*B, iNOS, and COX-2) and Healing Growth Factors (VEGF and TGF-*β*) Proteins

**Figure 8:**
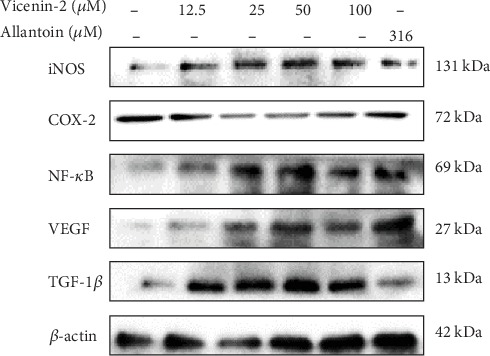
The effect of VCN-2 on the expression of NF-*κ*B, iNOS, COX-2, VEGF, and TGF-1*β* in HDF cells. A density of 5 × 10^5^ cells/well of cells were seeded in 6-well plates and treated with indicated concentrations of synthetic drugs compound for 24 h. The protein of cells was collected through RIPA buffer and analyzed by western blotting. *β*-actin have been acted as standard loading control to determine the target protein molecules.

**Figure 9:**
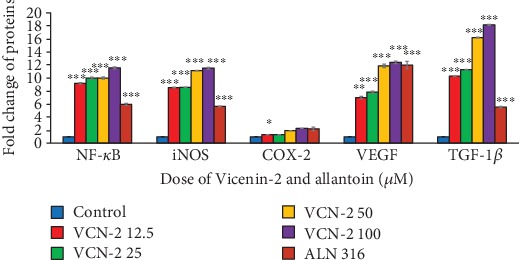
Effect of VCN-2 on the expression targets NF-*κ*B, iNOS, COX-2, VEGF, and TGF-*β* in HDF cells. The protein of cells was collected at 24 h through RIPA buffer after VCN-2 challenge and analyzed by western blotting. Cumulative value is expressed as mean ± SEM. ^∗∗∗^*p* < 0.001, ^∗∗^*p* < 0.01, and ^∗^*p* < 0.05 vs. control group without VCN-2, *n* = 3.

Immunoblotting was performed to investigate the expression and activities of inflammatory mediators and wound healing growth factors of HDF cells after treated with VCN-2 synthetic compound in specified concentrations ranged from 12.5 to 100 *μ*M. As illustrated in Figure 8, the inflammatory mediators including NF-*κ*B, iNOS, and COX-2 and wound healing growth factors comprising of VEGF and TGF-*β* proteins were evaluated in this study. Data in Figure 9 indicated that all of the mediator and growth factor proteins have markedly expressed with the treatment of allantoin and VCN-2 in dose-dependent manner as compared to the control untreated group. VCN-2 (100 *μ*M) treatment resulted the highest expression for all involving proteins with the relative density fold change of NF-*κ*B (11.55 ± 0.15), iNOS (11.49 ± 0.14), COX-2 (2.27 ± 0.01), VEGF (12.38 ± 0.20), and TGF-1*β* (18.12 ± 0.06).

### 3.6. Determination of Single-Layer Blank Hydrocolloid Films Texture with Different SA Percentage

**Figure 10:**
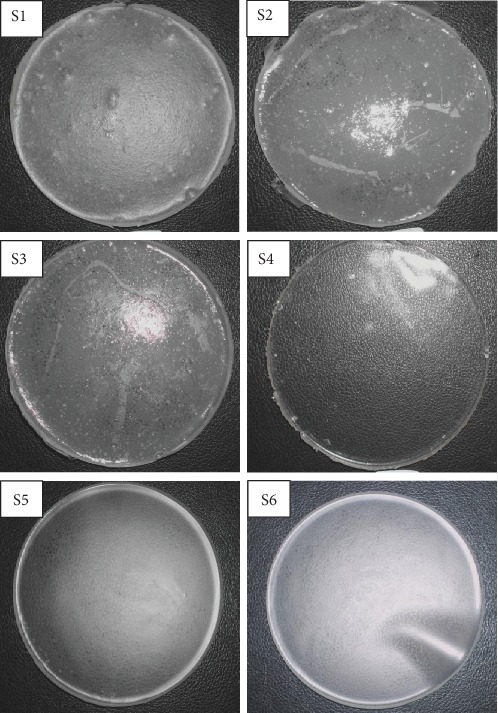
The developed blank SA hydrocolloid films in the pre-formulation study. The blank film made by 10% SA had been known as S1 formulation, S2 formulation 8% SA, S3 formulation 6% SA, S4 formulation 4% SA, S5 formulation 4.5% SA, and S6 formulation 4.5% SA with extra 4% gelatin powder as ingredient. Each experiment was carried out in triplicate.

Pre-formulation study is an important preliminary study to verify the goal of designing an optimum drug delivery system before the development of a new pharmaceutical formulation. In this study, physical evaluation including flexible, homogeneous, and smooth surface of hydrocolloid film were used to determine an ideal wound dressing synthesize. Results in Figure 10 showed that blank hydrocolloid films made with higher concentrations (10, 8, and 6% (w/w)) of SA produced lumps on the surface of films. Meanwhile, the concentrations of 4.5 and 4% (w/w) of SA polymer as a base for the hydrocolloid film (Figure 10) were found to be free of dissolved lumps and were more homogeneous and smoother. However, the films were found to easily break during the handling process. A result from Figure 10 also suggested S6 is the optimum formulation because film made from the ingredients 4.5% SA, 15% propylene glycol, 6% glycerol, and 4% gelatin were found to be more flexible, easily flow with no entrapped air bubbles, and can be removed from a container without breaking.

### 3.7. Development Hydrocolloid Films Permeated with VCN-2 and ALN

**Figure 11:**
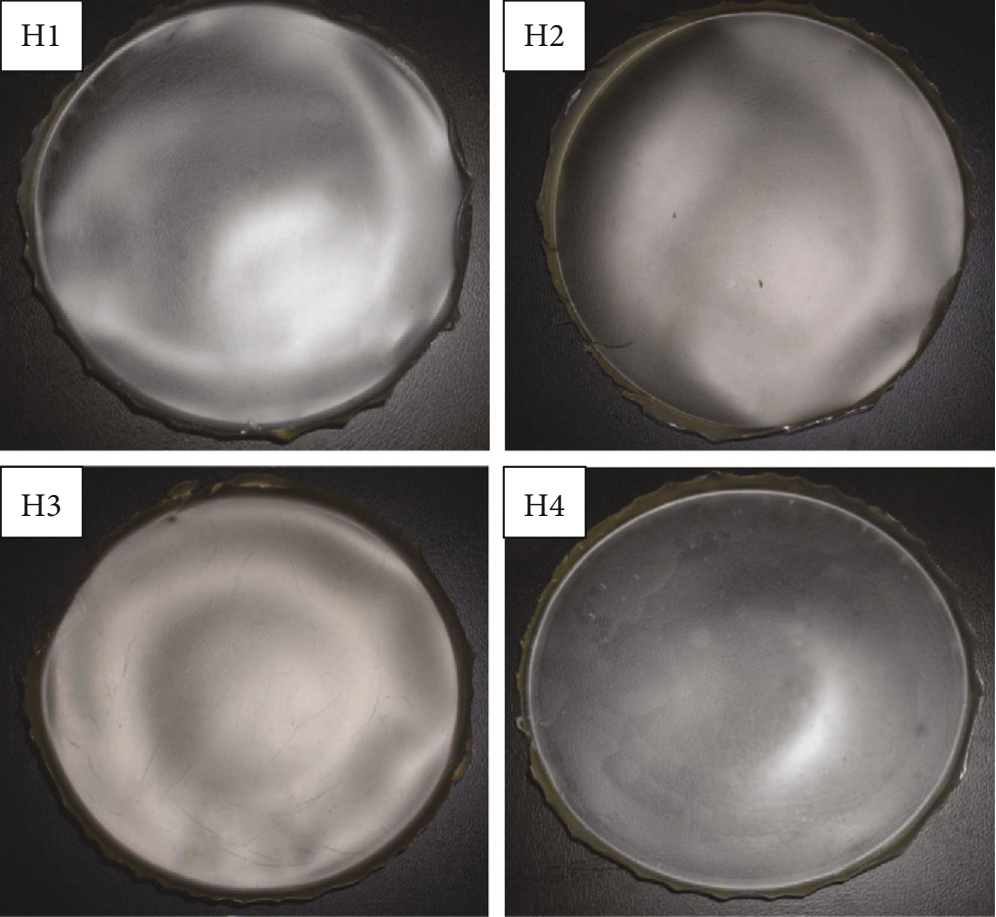
The image represented the developed new formulation permeated with drugs. H1: blank hydrocolloid films which made of 4.5% SA, 10% propylene glycol, 4% glycerol, and 4% gelatin. H2: The films incorporated with drugs such as 12.5 *μ*M VCN-2, H3: 25 *μ*M VCN-2. H4: 316 *μ*M allantoin, (*n* = 3).

Based on the physical appearance evaluation, all newly developed hydrocolloid films (20 g gel prior drying) including blank or drug-loaded formulations were pliable and retained smooth surface. All films showed a semitransparent texture surface especially for those impregnated with VCN-2, with the translucence increasing in dose-dependent manner as compared with blank film. Moreover, the film impregnated with allantoin gave a slight opaque, yellowish-brown appearance. The film loaded with allantoin had a less translucent appearance as compared to films loaded with VCN-2 (Figure 11).

### 3.8. Analysis of Thickness, Solvent Loss, and pH of Hydrocolloid Films

The thickness of all hydrocolloid film was shown in Table 3. The blank film with 0.137 ± 0.005 mm was the thinnest film, whereas the film loaded with 316 *μ*M allantoin, 0.175 ± 0.006 mm, was the thickest film in this study. On the other hand, the thickness of hydrocolloid films impregnated with VCN-2 increased in accordance to the dosages of VCN-2 added into film.

Besides, all developed film dressings had undergone solvent loss from hydrogel through drying process (Table 3). In this study, we observed that solvent loss of film was correlated with thickness of film. The higher the quantity of solvent loss, the thinner the film forming after the drying process. It showed that all film solutions exhibited pH values in the acidic range. The pH varied from 5.82 to 5.86. Addition of VCN-2 and allantoin drugs demonstrated lower pH value as compared to blank film (pH = 5.86).

### 3.9. Determination of Water Vapor Weight Gain per Area of Hydrocolloid Film

**Figure 12:**
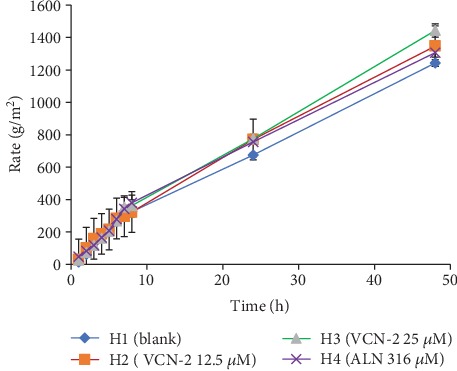
The weight gain of water vapor per area of the blank hydrocolloid film and hydrocolloid films which were impregnated with different concentrations of VCN-2 and allantoin. The data is expressed as mean ± SEM, *n* = 3. MVTR for the films for 48 h were derived from the slope gradient.

An ideal wound dressing needs an optimum rate of moisture vapor transmission rate (MVTR) to control water loss from skin through evaporation.  Figure 12 indicated the MVTR value for blank developed hydrocolloid film is 674.94 ± 131.6 g/m^2^/day. The MVTR value of hydrocolloid films containing VCN-2 and allantoin is higher as compared to blank film. The 12.5 *μ*M VCN-2, 25 *μ*M VCN-2, and 316 *μ*M allantoin hydrocolloid films have the MVTR value of 770.16 ± 151.1, 774.89 ± 149.9, and 756.61 ± 148.4 g/m^2^/day individually on 24 h. Besides, Figure 12 also showed all of the films had steadily increased MVTR reading over 48 h.

### 3.10. Detection Expansion Rate of Hydrocolloid Film

**Figure 13:**
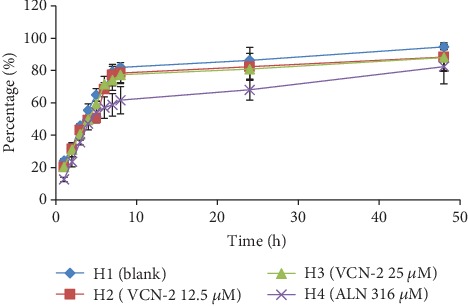
The expansion percentage of blank, 12.5 *μ*M VCN-2, 25 *μ*M VCN-2, and 316 *μ*M allantoin developed hydrocolloid film. Triplicate data is expressed as mean ± SEM.

Percentage of a film expansion (swelling) was closely related with the drug release patterns since it played an important role in controlling drug release from the dressing [15]. In this study, gelatin (4%) was used to represent a wound site for expansion rate study of all developed hydrocolloid films in exudation condition.  Figure 13 showed that all of the developed films had a rapid increase in percentage of film expansion within first 8 h. The swelling percentage then slowed down and expanded uniformly from 8 to 24 h. On 24 h, swelling percentage of blank, 12.5 *μ*M VCN-2, 25 *μ*M VCN-2, and 316 *μ*M allantoin hydrocolloid films were correspondingly indicated by 86.33 ± 13.7, 82.25 ± 14.5, 80.86 ± 10.6, and 68.03 ± 10.9%. The expansion percentage was nearly close to plateau for 24–48 h. The disc-shaped film on gelatin substrate had completely turned into a gel with large diameter in after more than 48 h.

### 3.11. Analysis Mechanical (Tensile) and Rheological Properties of Developed Film

Mechanical properties of developed hydrocolloid films were evaluated through tensile strength and elongation at break. Based on the result on Table 4, drug-loaded films (H2-H4) by VCN-2 and allantoin expressed higher tensile strength and lower elongation at break than the control blank hydrocolloid film (H1).

**Figure 14:**
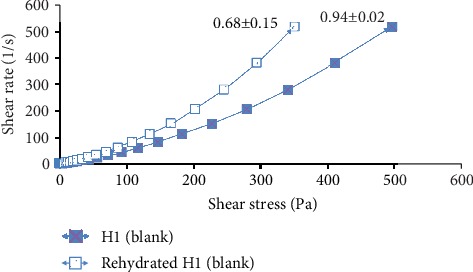
Rheology profile of gel and rehydrated gel of developed blank hydrocolloid film. The data shown at the apex of flow curve are the average of three replications of apparent viscosities (Pa s) at 500 s^−1^.

**Figure 15:**
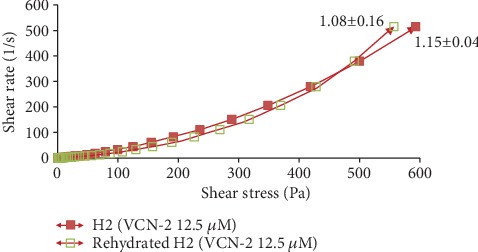
Rheology profile of the gel and rehydrated gel of 12.5 *μ*M VCN-2 film. The data shown at the apex of flow curve are the average of three replications of apparent viscosities (Pa s) at 500 s^−1^.

**Figure 16:**
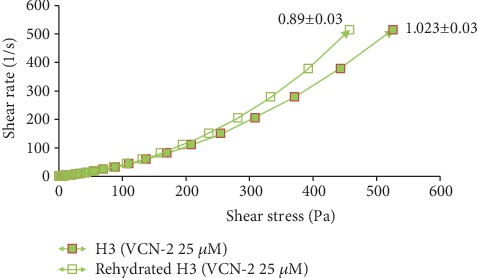
Rheology profile of the gel and rehydrated gel containing 25 *μ*M VCN-2. The data shown at the apex of flow curve are the average of three replications of apparent viscosities (Pa s) at 500 s^−1^.

**Figure 17:**
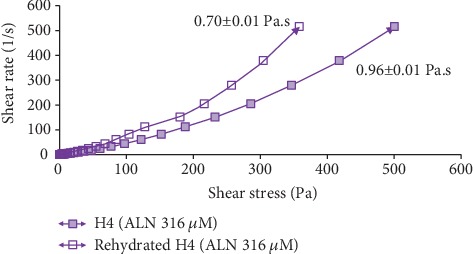
Rheology profile of allantoin gel and rehydrated gel at concentration 316 *μ*M. The data shown at the apex of flow curve are the average of three replications of apparent viscosities (Pa s) at 500 s^−1^.

According to the study of Ng et al. [16], rheological study has been performed by comparing the rheological properties of gel (before drying) and the rehydrated film through the film's flow curve of shear rate at 500 s^−1^ against shear stress. In this study, all the drug-loaded hydrocolloid films by VCN-2 or allantoin have higher apparent viscosities as compared to blank film (Figures 14–17). As seen in Figure 14, the blank film value was 0.94 ± 0.02 Pa s. The 12.5 *μ*M VCN-2 film presented 1.15 ± 0.04 Pa s (Figure 15), 25 *μ*M VCN-2 film displayed 1.02 ± 0.03 Pa s (Figure 16), and 316 *μ*M allantoin film indicated 0.96 ± 0.01 Pa s (Figure 17). All of the rehydrated gels possessed lower viscosity values as compared to developed gels of hydrocolloid film before drying.

### 3.12. Determination Drug Release of Hydrocolloid Film

**Figure 18:**
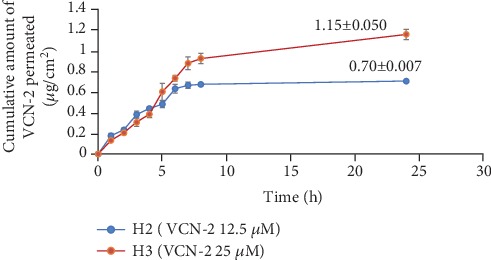
Cumulative amount of 12.5 and 25 *μ*M of VCN-2 release profile from a model hydrocolloid film wound dressing through Franz diffusion cell method. Three replication experiments were carried out and experimental data expressed as mean ± SEM.

The drug release profile of VCN-2 compound from developed hydrocolloid film was performed through the Franz cell apparatus and the drug released was assessed over a 24 h period, assuming that the film dressings were applied as once daily dressing. The drug release plot was shown in Figure 18, and results indicated that VCN-2 hydrocolloid films released drug steadily at initial 8 h. Finally, the total drug release is in dose-dependent manner from 12.5 *μ*M and 25 *μ*M VCN-2 films with values 0.70 ± 0.007 *μ*g/cm^2^ and 1.15 ± 0.050 *μ*g/cm^2^, respectively, at 24 h.

## 4. Discussion

MTT assay is entailed for the conversion of MTT tetrazolium salt to an insoluble formazan product, followed by quantification of solubilized formazan by spectrophotometry [17]. In this study, MTT assay was performed to evaluate the viability of HDF cells treated with VCN-2. The result in Figure 1 indicated that the cells remained feasible even after treatment with VCN-2 with concentration up to 400 *μ*M. The 24 h cytotoxicity assay presented prospective result showing no significant cytotoxicity effect on the HDF cells with the cell viability maintaining above 90%. Our finding is consistent with the study of Ku and Bae [18], which reported that treatment with VCN-2 on human umbilical vein endothelial cells (HUVEC) cells for 24 h did not affect cell viability, evident by cell viability percentage more than 100%. Hence, VCN-2 with no significant side effect on HDF cell was utilized in subsequent topical prototype development.

Meanwhile, the effect of VCN-2 on HDF on cell proliferation was assayed through CCK-8 kits and assessed using spectrophotometry. The result demonstrated that treatment with VCN-2 at low concentration (6–25 *μ*M) has significantly enhanced the cell proliferation while at higher concentration (50–400 *μ*M) reduced cell proliferation in a dose-dependent manner (Figure 2). We suggested this occurrence might be due to biphasic characteristic of VCN-2. For example, in low concentrations (0.01, 0.1, and 1 *μ*g/mL) of VCN-2 obtained from *U. circularis*, it has given a potential anti-inflammatory effect to inhibit the secretion of pro-inflammatory cytokines and mediators of LPS-induced macrophages. In contrast, VCN-2 exerted pro-inflammatory activity in high concentrations (10, 100, 500 *μ*g/mL) [19].

A standard scratch wound healing assay was performed to detect the spreading and migrating capability of HDF cells incubated with VCN-2. Present study showed there was correlation between the upregulation of cell viability and cell migration, where higher rate of cells viability supported and showed better improvement in wound closure rate (Figures 3–5). Data obtained suggesting VCN-2 may exhibit wound healing action by promoting HDF cells synchronized proliferate and migration. This finding showed similar phenomena with our previous study of Muhammad et al. [10, 11], which VCN-2 in MO fraction accelerated *in vitro* fibroblast proliferation and migration.

Furthermore, in ELISA quantification assay, VCN-2 exhibited a dose-dependent stimulatory effect on pro-inflammatory cytokines (TNF-*α*, IL-6, and IL-1*β*) level (Figures 6 and 7). The presence of TNF-*α*, IL-6, and IL-1*β* cytokines is very vital at early phase of healing as it stimulate wound restoration [20]. According to a study by Chin et al. [21], MO leaf extract film at dosage 0.1 and 0.5% successfully upregulated the production of TNF-*α* and IL-6 in abrasion and excision wound of diabetic Sprague Dawley rats. Whereas, at a higher concentration (50–100 *μ*M) of VCN-2 treatment, a significant rise of pro-inflammatory cytokines level was observed in the current study (Figures 6 and 7). This observation has insinuated that the overproduction of pro-inflammatory cytokines might lead to prolong inflammation and delay the proliferation phase during wound healing. This finding was validated in the study of Xie et al. [22] and Zhang et al. [23], where excessive production of TNF-*α* and IL-1*β* impede wound healing through stimulated MMP expression and further exacerbate breakdown of tissues.

There is a similar modulation effect of VCN-2 compound on pro-inflammatory mediators' expression (Figure 8) of HDF cells through immunoblot assay assessment. Data in Figure 9 showed an increased expression of NF-*κ*B, iNOS, and COX-2 in concentration-dependent fashion. As mentioned earlier, the existence of these mediators played important roles during the inflammatory phase of wound healing to recruit wound healing cells in local wound environment [24]. However, there was an excessive expression of these mediators at the higher concentration of 50–100 *μ*M VCN-2 as shown in Figure 9. We propose that such results might reflect the reasons of delaying inflammation reaction which lead to further deterioration effects on wound. The present results agreed with those reported by Choi et al. and Vo et al [25], they found that inflammation occurs due to stimulation of NF-*κ*B, iNOS, and COX-2; therefore, inhibited activation of these pro-inflammatory mediators have attenuated adverse inflammation related illness.

Moreover, growth factors such as VEGF and TGF-*β* expression as shown in Figure 8 was evaluated through immunoblot protein qualification. Both expressions of VEGF and TGF-*β* of HDF cells were enhanced in response of VCN-2 treatment (Figure 9). During the wound healing process, VEGF acts to induce cells proliferation and migration through angiogenesis while TGF-*β* stimulates fibrogenesis and remodeling through differentiation and development [26]. Studies of Zhang et al. [27, 28] verified that acceleration healing rates of human skin fibroblast cell line (Hs27) is own to the presence of VEGF and TGF-*β* following application of herb *Astragali Radix* (AR) and *Rehmanniae Radix* (RR).

**Figure 19:**
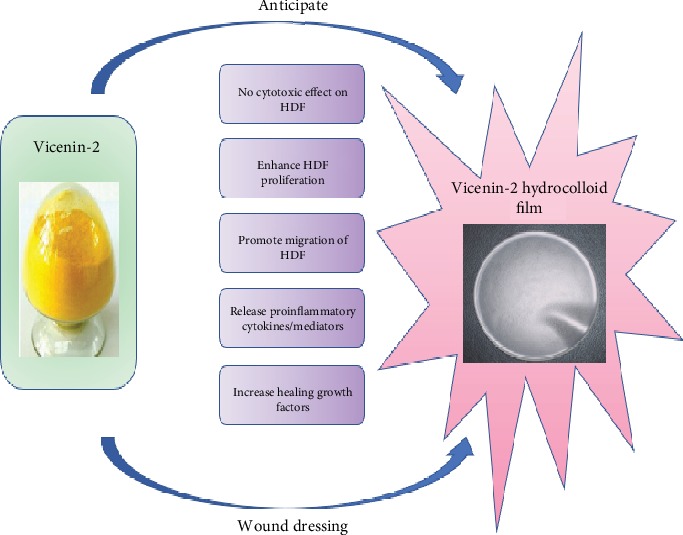
Characteristics exerted by VCN-2 compound on human dermal fibroblast cells showed that it might enhance wound healing. Abbreviation: human dermal fibroblast (HDF).

Current *in vitro* results indicated that synthetic VCN-2 compound at lower dosages (12.5–25 *μ*M) gives optimum healing properties. As shown in Figure 19, VCN-2 showed no significant toxic effect to HDF while promoting the cell proliferation and cell migration. In addition, it releases low levels of pro-inflammatory cytokines and mediators to avert the prolonged inflammation, which is common in chronic wound. But it enhanced wound healing by producing VEGF and TGF-*β* in wound sites. Therefore, 12.5–25 *μ*M VCN-2 is utilized in subsequent new wound dressing formulation.

A rational dosage for new medical formulation development starts with pre-formulation studies on physical and chemical properties of the drug substance alone or in combination with excipient pharmaceutical ingredients [29]. Figure 10 revealed that a higher percentage of SA produced dressing with lumps. The presence of lumps is due to the polymer powders not completely dissolved in final gels prior to drying and this might affect the drug delivery later on. According to Kianfar et al. [30], lumps arising on final wound dressing is one of the defect criteria in film formulation due to insolubility of the alginates.

The co-solvents such as propylene glycol and glycerol are added to overcome this problem [31]. The propylene-glycol gives a moisturizing effect, while glycerol played a vital role to increase the softness and flexibility of films by increasing the distance between the polymer chains [32]. In addition, gelatin has exerted good biocompatible and biodegradability characteristics and offer a moist environment which enhances wound by absorbing wound exudates. In this study, hydrocolloid film with gelatin has increased in tensile strength and elongation at break (EAB). This result is in accordance with the investigation of Syarifuddin et al. [33]. Thus, among the films which were made from SA (4–4.5% (w/w)) in Figure 10, S6 was the satisfactory development formulation with the minimum amount of ingredients required to form an ideal VCN-2 hydrocolloid films that were clear with no lumps and possess flexibility to cover the wound for a long period of time.

As shown in Figure 11, incorporation of VCN-2 and allantoin resulted in lower transparency in hydrocolloid film as compared to blank film. This occurrence might be due to drug compound interaction with SA or gelatin in developed film which can further lead to less visible light transmission through film. This appearance of film in this study agreed with a previous report from Prodpran et al. [34]: gelatin hydrocolloid film exhibited translucent texture and shown low light transmission. Besides, allantoin film in this study displayed slightly yellowish-brown colour (Figure 11). The colour of film was resulted from the pigment possessed by allantoin. The study of Denavi et al. [35] revealed that intense yellowish colour of composite film was increased proportional with concentration soybean protein added into film.

In this study, the thickness of a developed film was observed inversely correlates with the solvent loss of film (Table 3). As seen, the thickness of a hydrocolloid film was slightly reduced by increasing the amount of solvent loss. Polymer coagulation of films occurred due to water solvent loss through evaporation [2]. Besides, the thickness of a hydrocolloid film was slightly increased after being incorporated with medicinal drug compound such as VCN-2 and allantoin as compared with blank film (Table 3). This occurrence might be due to a great amount of solids from the drug compound present in the film. A similar finding was also given by Rezvanian et al. [6]; addition of simvastatin drug compound has increased thickness of alginate/pectin composite film.

Gethin [36] had reported that acidic wounded site (low pH) has played an essential role to enhance wound healing through oxygen release, angiogenesis, protease activity, and bacterial toxicity. Otherwise, the impaired wound has exhibited elevation of alkaline environment (pH 7.15–8.9). Interestingly, pH measurement of all developed hydrocolloid gels which have dissolved in distilled water has displayed acidic values (Table 3) due to the interaction of chemical composition and microstructure of dressing to release alginic acid [37]. A similar pH ranged (4.95–6.91) of several alginate, carboxymethylcellulose hydrocolloid dressings were also reported by Uzun et al. [38]. Therefore, hydrocolloid film (acidic) in this study might promote wound healing own to its hydrophilic exudation absorb properties to reduce the liquid retaining (neutral or mildly alkaline) in wound site.

According to Gupta et al. [39], MVTR for normal skin is 204 g/m^2^/day but it ranges from 279 to 5138 g/m^2^/day for injured wound. In the current study, MVTR for all developed films fall within the range for wound skin, which is 279–5138 g/m^2^/day (Figure 12). This proposed that the developed film was permeable to aqueous vapor, was able to provide a humid condition to enhance cell migration for macrophages and fibroblasts, and was able to facilitate the transportation related cytokines, mediator, and growth factors into wound sites [40]. As shown in Figure 12, the MVTR value of all films was upregulated over the 48 h to allow an optimum transmission and gases exchange. A previous study of Xu et al. [41] have stated that controlled optimum MVTR of wound dressing within (≤2028 g/m^2^/day) was able to create a desirable milieu, which is essential for wound recovery by prevented accumulation of wound exudates and excessive dehydration at wound site.

Gelatin gel (4%) is an ideal model to represent suppurating wound in human due to its ability to enhance humid condition [42]. Developed hydrocolloid film absorbed the moisture and underwent expansion to allow VCN-2 compound to be released from the dressing.  Figure 13 presented that adding drug compound in film have reduced the swelling percentage of VCN-2 and allantoin hydrocolloid film. This result suggested that drug-loaded film might be desirable for heavy exudative wound in a longer period. It allows the drug compound in the film to have sustained-release into wound site. This finding is supported by the study of Purwar et al., [43] and Sarheed et al. [44]; releasing of BSA drug from composite hydrogel and metronidazole drug from alginate hydrogel were attributed to swelling behavior of dressing.

As one of the acceptance criteria defined, all the developed dressing exhibited interesting behavior with respect to its tensile strength and percentage elongation at break. From the result of Table 4, the developed hydrocolloid films with the presence of drugs showed greater tensile strength accompanies with relatively lower value of elongation at break via blank film. This feature explained that the developed films in this study possesses elasticity after the stretching of about 34.6–53.8% recovery in length. Similar findings were also given by Zaman et al. [45], Rezvanian et al. [6], and Rezvanian et al. [46], and it was found that tensile strength value of a dressing was decreased when there was a rise of elongation percentage.

Rheology study was important for the determination of plastic deformation stress of a film because it control the stability and efficacy of drug release [47]. It was conducted through flow measurement since prepared gel possesses viscoelastic properties and gel of rehydrated film possesses pseudoplastic flow behavior [48]. The present study revealed that addition of drugs such as VCN-2 or allantoin have attributed to a higher viscosity for the developed films (Figures 14–17). This suggested that the developed film has longer residence time on the wound site especially in suppurating wound accredited to its deformation resistant [42]. As reported by Khanbanha et al. [49], the higher the viscosity value of a hydrocolloid film dressing, the better the dressing at maintaining the integrity of a drug delivery device.

In this present study, it was observed that rate of 12.5 *μ*M VCN-2 release from developed film was high (77.4 ± 3.1%) at 8 h initially but it reduced into 40.4 ± 3.3% with the time passing up to 24 h (Figure 18). This data might explain that VCN-2 release from the film was influenced by the expansion of film which acts as drug carrier since the plotted graph pattern was linearly correlated with film expansion (Figure 13). A similar trend of drug release from composite dressing that was reported by Huang and Brazel [50] supported it by clarifying that highly release of drug initially provided immediate relief while slowly prolonged release drug subsequently further enhanced gradual healing. Besides, the drug release was relatively influenced by drug loading concentration. As mentioned, there was more amount of drug released from 25 *μ*M VCN-2 hydrocolloid film into the wound site as compared to 12.5 *μ*M VCN-2 at 24 h (Figure 18). This evidence was in agreement with previous report of Sriamornsak et al. [51] that 1.25% of theophylline released higher drug content from calcium pectinate gel bead as compared to 0.125% theophylline.

Collectively, wound dressing prepared using SA could absorb wound exudates and retain moisture at the wound site. VCN-2 hydrocolloid film based on SA was developed as a novel and suitable film-based drug delivery system intended to increase the efficacy of VCN-2 toward wound tissue. Thus, hydrocolloid film containing VCN-2 might accelerate the *in vivo* wound healing process.

## 5. Conclusion

In summary, *in vitro* results showed that a lower concentration of VCN-2 (12.5 *μ*M) gives better healing rate on HDF cells. Besides, hydrocolloid film recapitulated with concentration 12.5, 25, and 50 *μ*M of VCN-2 have been successfully developed in this study. VCN-2 hydrocolloid film could represent an alternative wound dressing, which holds a good promise in the future. One limitation of the current developed dressing is that it is not indicated for multiple-type infected wounds due to it being only consisted of a single drug in it. Further advanced studies which focus on clinical testing are needed to assess for fully understand the safety, clinical, and economic benefits of this new dressing technology.

## Figures and Tables

**Table 1:** Components of drug-free (blank) single-layer hydrocolloid films development in pre-formulation study.

Formulation	Sodium	Gelatin (g)	Propylene	Glycerol (g)	Distilled
	Alginate (g)		Glycol (g)		Water (g)
S1	10	—	15	6	69.0
S2	8	—	15	6	71.0
S3	6	—	15	6	73.0
S4	4	—	15	6	75.0
S5	4.5	—	15	6	74.5
S6	4.5	4	15	6	70.5

**Table 2:** Ingredients to develop new hydrocolloid films formulation which compromise VCN-2 and allantoin compound.

Formulation	Sodium alginate (g)	Drug compound-loaded X 10 ^−4^ (g)	Gelatin (g)	Propylene glycol (g)	Glycerol (g)	Distilled water (g)
H1	4.5	—	4	15	6	70.500
(Blank)						
H2	4.5	VCN-2	4	15	6	70.499
(VCN-2 12.5 *μ*M)		12.5				
H3	4.5	VCN-2	4	15	6	70.498
(VCN-225 *μ*M)		25.0				
H4	4.5	ALN	4	15	6	70.495
(ALN316 *μ*M)		50.0				

VCN: Vicenin; ALN: allantoin.

**Table 3:** The physico-chemical characteristics of developed SA hydrocolloid films consisting of VCN-2 and allantoin.

Formulation	Thickness (mm)	Solvent loss (g)	pH
HI (blank)	0.137 ± 0.005	17.251 ± 0.024	5.86 ± 0.006
H2 (VCN-212.5 *μ*M)	0.148 ± 0.007	17.223 ± 0.170	5.83 ± 0.005
H3 (VCN-2 25 *μ*M)	0.154 ± 0.005	17.126 ± 0.044	5.82 ± 0.003
H4 (ALN 316 *μ*M)	0.175 ± 0.006	16.486 ± 0.108	5.83 ± 0.007

VCN-2: Vicenin-2; ALN: allantoin.

**Table 4:** The tensile strength and elongation at break of developed hydrocolloid films dressing consisting of VCN-2 and allantoin.

Formulation	Tensile strength (N/mm^2^)	Elongation at break (%)
HI (blank)	2.417 ± 0.05	53.8 ± 1.2
H2 (VCN-2 12.5 *μ*M)	2.808 ± 0.30	46.5 ± 3.1
H3 (VCN-2 25 *μ*M)	2.978 ± 0.45	38.3 ± 0.4
H4 (ALN 316 *μ*M)	2.422 ± 0.10	34.6 ± 5.0

VCN-2: Vicenin-2; ALN: allantoin.

## Data Availability

The data used to support the findings of this study are available from the corresponding author upon request.
